# Novel glycosyl prodrug of RXP03 as MMP-11 prodrug: design, synthesis and virtual screening

**DOI:** 10.1186/s13065-023-01075-1

**Published:** 2023-11-25

**Authors:** Moaz M. Abdou, Ferenc Ötvös, Dewen Dong, Magdalini Matziari

**Affiliations:** 1 Egyptian Petroleum Research Institute, P.O. 11727, Nasr City, Cairo Egypt; 2 Institute of Biochemistry, Biological Research Centre, Hungarian Academy of Sciences, 6726 Szeged, Hungary; 3 Changchun Institute of Applied Chemistry, Chinese Academy of Sciences, Changchun, 130022 China; 4 Department of Chemistry, Xi’an Jiaotong Liverpool University, Suzhou, 215123 Jiangsu People’s Republic of China

**Keywords:** Prodrug, **RXP03**, Phosphinic ester, Matrix metalloproteases, Molecular docking, Permeability enhancer

## Abstract

Like most phosphinic acids, the potent and selective **RXP03** inhibitor of different MMPs exhibited moderate absorption and low bioavailability, which impaired its use. In an unprecedented attempt, we present an interesting synthetic approach to a new class of phosphinate prodrug, glycosyl ester of **RXP03**, to provide a potentially improved blood–brain barrier (BBB) behavior compared to the former lead compound **RXP03**. To validate this speculation, a predictive study for permeability enhancer of glycosyl ester of **RXP03** showed encouraging insights to improve drug delivery across biological barriers.

## Introduction

**Fig. 1:**
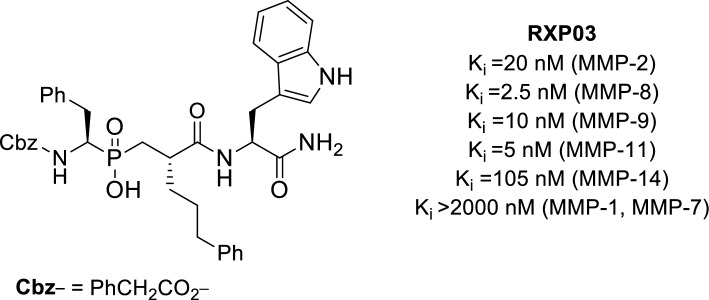
**RXP03** is an effective inhibitor of (MMPs)

There is growing evidence that matrix metalloproteases (MMPs) are potential targets for cancer therapy [1, 2]. It has been found that stromelysin-3 (MMP-11), a member of the MMP family that acts as a survival factor on cancer cells rather than as an inducer of cancer cell proliferation, is mainly involved in the formation of tumors rather than the growth of them [3–5]. The phosphinic peptide **RXP03** (Fig. 1), which contains an unusually long side chain at P1’, was highly effective against various matrix metalloproteinases [6–9].

Unfortunately, this compound’s low lipophilicity and poor membrane permeability have prevented clinical application. A prodrug approach is proposed to achieve this goal by improving its absorption properties. The prodrug approach can mask the ionizable hydroxyl group in **RXP03** to increase its lipophilicity and decrease salt formation. After absorption and enzymatic hydrolysis, the prodrug could release the active drug into the cells [10].

**Fig. 2:**
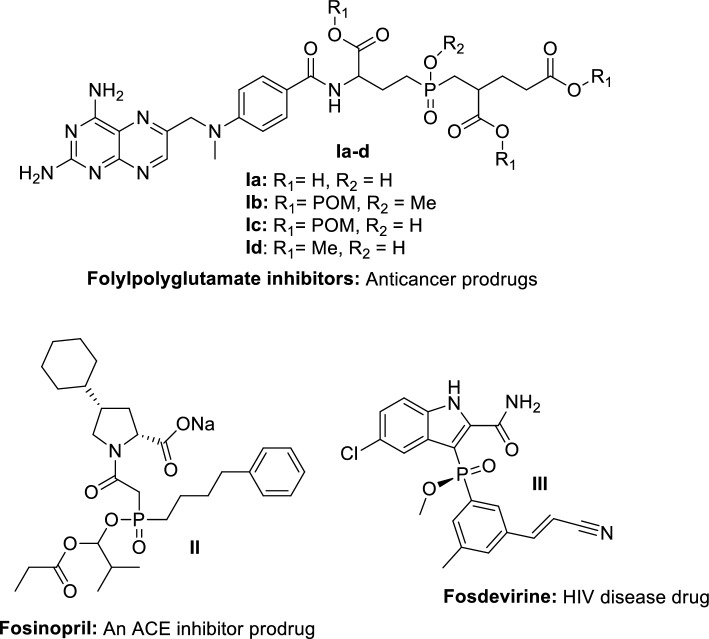
Phosphinic acid prodrugs and drugs with potential clinical use

Choosing an attached core to incorporate into this type of prodrug was conceptually appealing as a starting point for the design [11–13]. Even though many compounds have interesting biological activities, a literature review prompted us to think that conjugating drugs with sugar units, especially glucose, might be an interesting aspect of the prodrug/derivatization approach. Several conjugates are more effective than their parent drugs at drug delivery [14–16]. As another feature of the attachment of sugar, glucose might facilitate transport pathways across multiple biological barriers [11–13]. As a result, sugar conjugation is capable of (i) using active transport systems, (ii) modifying the construct’s physical properties, and (iii) transporting to a specific target [11–13]. Notably, the absorption of *β*-linked sugar-drug conjugates is significantly higher than the *a*-anomers, which was the main reason for choosing 1,2,3,4-*tetra*-*O*-acetyl-*β*-d-glucopyranose (TGA) as the sugar component linked with **RXP03** [17]. Glucose byproducts from the cleavage would not be toxic, and the resultant drug would not be stereogenic to phosphorus. Interestingly, several phosphinate esters have been developed and used in clinical trials (Fig. 2) [18–23], but never sugar-based esters as proposed here.

Recently we have been interested in enhancing the esterification reaction of phosphinic acids. In the light of these findings and continuation of our prior work on the synthesis of highly selective inhibitors of MMP-11, such as **RXP03** [24, 25], we reported the design and synthesis of a novel class of phosphinate prodrug derived from glycosyl ester (Glycosyl prodrug of **RXP03** according to our esterification method [26] with the vision of improving the penetration of the BBB, followed by studying the tissue distribution of both parent-drug **RXP03** and its prodrug, in particular, crossing the BBB using molecular docking studies.

## Results and discussion

### Chemistry

**Scheme 1:**
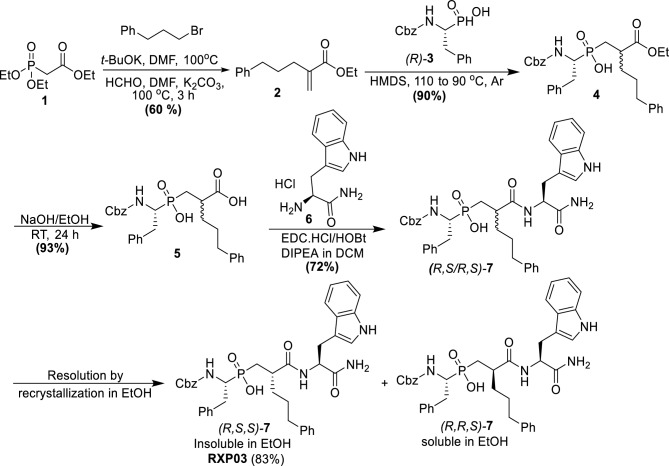
The synthetic pathway used to prepare (*R, S, S*)*-7* and the resolution of its diastereomers

The diastereoselective synthetic protocol presented in Scheme 1 allows the synthesis of the two diastereoisomers of **RXP03** in gram scale and high yields using our previously reported synthesis protocol (Scheme 1) [24]. The required ethyl 2-methylene-5-phenylpentanoate **2** was synthesized by alkylation of triethyl phosphonoacetate **1** with 1-bromo-3-phenylpropane followed by a Horner–Wadsworth–Emmons (HWE) condensation with formaldehyde. Michael-type addition of acrylate **2** to (*R*)-Z-PhePO_2_H_2_ **(*R*)-3** by activation with HMDS leads to the phosphinic dipeptide **4**. Saponification of the ethyl ester of **4** produced **5**. Coupling with (*S*)-TrpNH_2_ **6** provided the phosphinic pseudo tripeptide *(R, S/R, S)-****7*** as mixture of pairs of isomers with different solubility properties. When *(R,S,S/R,R,S)-****7*** was treated with absolute ethanol, a solid precipitated, which proved to be isomer *(R, S, S)-****7***, while the filtrate consisted exclusively of isomer *(R, R, S)-****7***. Finally, the purity and assignment of the absolute configuration of the target isomer *(R, S, S)-7* were performed according to methods described in detail previously using RP-HPLC [24].

**Scheme 2:**
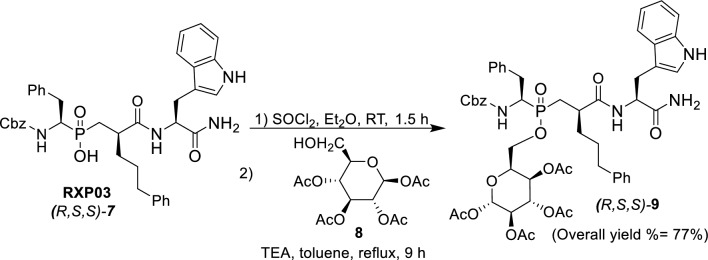
Synthesis of target glycosyl prodrug **9**

The prodrug **9** was prepared following our previous procedure [26] via the phosphinic chloride, which was generated by treatment of the phosphinic acid **RXP03** with thionyl chloride in anhydrous diethyl ether; the phosphinic chloride was then reacted in situ with 1,2,3,4-*tetra*-*O*-acetyl-*β-d*-glucopyranose **8** in the presence of a catalytic amount of triethylamine to give the desired glycosyl prodrug **9**, as shown in Scheme 2.

### Virtual screening

The docking energies, ligand efficiency (LE) values, and the list of the contacting amino acid residues are summarized in Tables 1, 2, 3. As can be seen, the docking energies within the glycosylated-unglycosylated pairs of molecules hardly differ, which is not feasible regarding the huge structural difference within each pair. This is the consequence of the additive energy calculation method of the docking programs. To scale down this additivity, different ligand efficiency values are used; here, the docking energy was divided by the number of non-hydrogen atoms of the ligand [27]. As a consensus, higher than − 0.24 a ligand is considered a weak binder or non-binder. The comparative interaction patterns for this series of ligands are shown in Tables 1, 2, 3. For comparison, the putative binders apatinib and (−)-epigallocatechin-3-gallate (EGCG) were also involved in the docking calculations.

**Table 1:** The free energy estimates and molecular docking interactions of investigated compounds towards Claudin-4

Compound	Docking score (Kcal mol^−1^)	LE	Binding sites
Apatinib	− 9.02382	− 0.3008	LEU70, PHE35, ASP68, GLU48, ALA72, LEU73, TYR67, SER69, ARG158, ARG81, ILE46
EGCG	− 9.3447	− 0.2832	TYR67, ARG81, ALA72, LEU73, PRO74, ASN53, GLN78, ARG158
RXP03_RRR	− 14.37098	− 0.2874	LEU70, ARG81, LYS65, ASP68, GLU48, PRO74, ALA72, LEU73, GLN78, TYR67, ASN53, ARG158
RXP03_RRR_gluc_Ac	− 16.31292	− 0.2235	ASP68, TYR67, ALA72, LYS65, PRO74, ARG158, LEU70, ARG81, VAL55, ASN53, LEU73, GLN63, ILE46, SER69
RXP03_RRS	− 14.78712	− 0.2957	ASN53, PHE35, ALA72, VAL55, PRO74, LEU77, TYR67, LEU73, GLU48, GLN63, GLN44, GLN78, ILE40, ARG81, ILE46, LYS65, UNL1
RXP03_RRS_gluc_Ac	− 17.38592	− 0.2382	ASN53, PHE35, SER69, ALA72, GLN63, TYR67, LYS65, VAL55, PRO74, LEU73, ARG158, ILE46, THR33
RXP03_RSR	− 13.51703	− 0.2703	ARG81, VAL55, LYS65, LEU70, LEU73, ALA72, GLN63, TYR67, ASN53, SER69, ASP68
RXP03_RSR_gluc_Ac	− 15.76554	− 0.216	ALA72, GLU48, ARG158, THR33, TYR67, LYS65, ASP68, PHE35, PRO74, ARG81, GLN78, LEU73, SER69, ILE40, GLN156, UNL1
RXP03_RSS	− 14.18748	− 0.2837	VAL41, ARG158, LYS65, ILE40, PHE35, GLU48, ILE46, ASN53, VAL55, ASP68, GLN156
RXP03_RSS_gluc_Ac	− 16.16767	− 0.2215	ASN53, LYS65, PHE35, TYR67, ILE46, ARG158, ILE40, GLN156, VAL55, ASP68, VAL41, THR33, UNL1
RXP03_SRR	− 15.17881	− 0.3036	VAL55, PHE35, GLN156, LYS65, THR33, ARG158, TYR67, GLU48, ASP68, ILE40, GLN63, ASN53, GLN44
RXP03_SRR_gluc_Ac	− 16.7489	− 0.2294	GLN78, ASN53, TYR67, PHE35, ILE46, ARG81, ILE40, ARG158, ASP68, PRO74, VAL55, LEU70, ALA72, LEU73, LYS65, UNL1
RXP03_SRS	− 14.94215	− 0.2988	ILE46, VAL55, ILE40, GLU48, PHE35, LYS65, ARG158, ASP68, TYR67, GLN156, ASN53
RXP03_SRS_gluc_Ac	− 15.01769	− 0.2057	ARG158, ASN53, TYR67, LYS65, GLN156, VAL55, ILE46, ASP68, THR33, ILE40, GLN44, PRO74, PHE35, LEU73
RXP03_SSR	− 13.97376	− 0.2795	PHE35, ILE46, PRO74, ALA72, ASP68, LYS65, ASN53, TYR67, GLN63
RXP03_SSR_gluc_Ac	− 15.34149	− 0.2102	ASN53, TYR67, LYS65, ALA72, ARG158, ILE40, GLN44, THR33, GLN63, PHE35, PRO74, SER69, ILE46
RXP03_SSS	− 14.38745	− 0.2877	ASN53, PHE35, ILE46, VAL55, PRO74, ARG158, GLU48, TYR67, THR33, GLN156, LYS65
RXP03_SSS_gluc_Ac	− 17.19927	− 0.2356	ASN53, ARG158, LYS65, TYR67, LEU73, GLN44, ILE40, ALA72, PHE35, GLN63, PRO74, THR33, ASP68, ILE46, GLN156

**Table 2:** The free energy estimates and molecular docking interactions of investigated compounds towards Claudin-15

Compound	Docking score (Kcal mol^−1^)	LE	Binding sites
Apatinib	− 9.52239	− 0.3174	SER51, TRP63, ARG30, ILE75, LEU69, TYR74, ILE44
EGCG	− 7.68613	− 0.2329	ILE44, THR154, GLU46, ASP55, SER51, ALA53, TRP63, TYR156
RXP03_RRR	− 15.07626	− 0.3015	ARG30, PRO66, GLU46, TRP63, GLY153, LEU69, TYR151, SER51, TYR74
RXP03_RRR_gluc_Ac	− 14.8777	− 0.2038	SER51, ASN42, ILE44, TRP63, ASP55, TYR156, ALA53, THR154, THR41, CYS52, GLY153, GLU64, PRO66
RXP03_RRS	− 14.30466	− 0.2861	GLU64, ARG30, PHE65, PRO66, TYR151, SER51, TRP63, ILE44, GLY153, THR154, LEU69, ALA53, ILE75
RXP03_RRS_gluc_Ac	− 15.30323	− 0.2096	LEU69, ASN42, ILE44, TRP63, ALA53, THR154, SER51, PHE65, ASP55, ARG30, GLU64, GLY153
RXP03_RSR	− 15.81887	− 0.3164	GLU46, PHE65, THR154, ARG30, SER51, TYR74, LEU69, TYR151, TRP63, ILE75, TYR156, ALA53, ILE44
RXP03_RSR_gluc_Ac	− 17.51933	− 0.24	LEU69, ILE75, SER51, THR154, TRP63, PHE65, TYR151, ARG30, GLU64, GLY153, PRO66
RXP03_RSS	− 14.69353	− 0.2939	GLU46, GLY153, TYR74, PRO152, LEU69, SER51, TRP63, THR154, ARG30, ILE44, PHE65, TYR156, GLY73, ILE75, TYR151
RXP03_RSS_gluc_Ac	− 16.5613	− 0.2269	LEU69, ILE44, TRP63, PHE65, THR154, GLY153, TYR151, ARG30, SER51, GLU64, PRO152, PRO66
RXP03_SRR	− 15.34047	− 0.3068	TYR151, TYR156, LEU69, PHE65, ILE75, PRO66, SER51, TRP63, ARG30, ASP145, GLU46, PRO152, GLY153
RXP03_SRR_gluc_Ac	− 14.87264	− 0.2037	LEU69, ILE44, THR154, GLU64, TYR156, ALA53, PHE65, GLU46, TYR151, ARG30, PRO66, SER51, GLY73, PRO152, TRP63
RXP03_SRS	− 15.65649	− 0.3131	ARG30, GLY153, ASN141, ILE75, PRO66, THR154, GLU46, TYR74, PHE65, PRO152, TYR151, ASP145, SER51, TYR156
RXP03_SRS_gluc_Ac	− 16.91929	− 0.2318	LEU69, TYR74, ASP145, ASN42, ARG30, ALA53, TYR151, TRP63, TYR156, THR154, ILE75, SER51, ILE44, PHE65
RXP03_SSR	− 14.78361	− 0.2957	ARG30, ASN141, TYR74, ASP145, GLU46, SER51, PRO66, PHE65, TRP63, ARG144, LEU69, TYR151, ILE44, GLY153, TYR156, PRO152
RXP03_SSR_gluc_Ac	− 17.3547	− 0.2377	LEU69, TRP63, ARG30, GLU46, CYS52, SER51, TYR151, THR154, GLY73, PRO66, ILE75, PHE65
RXP03_SSS	− 15.85135	− 0.317	PHE45, SER32, TYR28, SER56, THR54, VAL31, THR43, ASN42, THR40, ILE44, PRO160
RXP03_SSS_gluc_Ac	− 13.96981	− 0.1914	LEU69, ARG30, TRP63, ASP55, TYR151, THR154, ALA53, PHE65, SER51, GLY153, TYR156, PRO66, ASN61, THR54

**Table 3:** The free energy estimates and molecular docking interactions of investigated compounds towards Claudin-19

Compound	Docking score (Kcal mol^−1^)	LE	Binding sites
Apatinib	− 8.52968	− 0.2843	ASN156, PRO154, VAL44, ILE40, ILE41, ALA157, TYR35, ALA55, THR42
EGCG	− 7.53282	− 0.2283	LYS65, TYR67, LEU46, GLU48, SER53, ALA55, GLN63
RXP03_RRR	− 14.91152	− 0.2982	LYS65, LYS31, TYR159, LEU143, TYR67, LEU73, GLU48, TYR35, PRO154, ASP74, ILE40, SER53, LEU46
RXP03_RRR_gluc_Ac	− 14.99871	− 0.2055	ILE40, VAL44, PRO154, ALA157, LYS31, TYR159, TYR35, TYR67, LEU46, LEU73, ASN156, GLU48
RXP03_RRS	− 15.44513	− 0.3089	TYR159, TYR35, ILE77, LYS31, ASP74, TYR140, LEU143, GLU48, VAL44, ILE40, TYR67
RXP03_RRS_gluc_Ac	− 16.34391	− 0.2239	ALA55, GLU147, TYR67, LEU73, GLU48, SER53, VAL44, LEU46, TYR35, ASP68, LYS65, ILE40, TYR140
RXP03_RSR	− 14.38601	− 0.2877	LEU143, TYR35, VAL44, GLU147, PRO154, TYR140, LYS31, ILE40, HIS76, ILE77, ALA157, ASN156
RXP03_RSR_gluc_Ac	− 15.06307	− 0.2063	THR42, LYS31, PRO154, TYR159, ALA157, TYR35, ALA39, LEU46, ILE41, ASN156, VAL44, SER56, ILE40, ALA55, UNL1
RXP03_RSS	− 15.10211	− 0.302	TYR159, TYR67, PRO154, TYR140, SER152, ASN156, LYS31, ALA157, LEU143, GLU48, ILE40, GLU147, ILE77, TYR35
RXP03_RSS_gluc_Ac	− 15.61777	− 0.2139	ALA55, THR42, LYS65, TYR159, LYS31, ILE41, ILE40, ALA39, LEU46, TYR35, VAL44, CYS64, GLN63
RXP03_SRR	− 15.78049	− 0.3156	LEU143, VAL44, LYS31, TYR67, PRO154, TYR140, TYR35, SER53, GLU48
RXP03_SRR_gluc_Ac	− 15.52744	− 0.2127	ALA55, THR42, LYS65, GLN63, VAL44, LEU46, SER56, ILE40, TYR35, ILE41
RXP03_SRS	− 16.6303	− 0.3326	TYR159, TYR35, VAL44, ILE77, PRO154, TYR67, LYS31, GLU48, LEU143, ALA157, ASN156, TYR140
RXP03_SRS_gluc_Ac	− 15.25153	− 0.2089	ALA55, TYR35, THR42, VAL44, LYS65, THR59, SER56, LEU46, ILE40, CYS64, GLN63, ILE41
RXP03_SSR	− 15.8194	− 0.3164	TYR35, VAL44, LYS31, TYR159, GLU48, TYR67, LEU143, HIS76, LEU46, ILE40
RXP03_SSR_gluc_Ac	− 16.48499	− 0.2258	LYS31, VAL44, LYS65, TYR159, TYR35, SER53, LEU46, TYR67, ALA55, GLU147
RXP03_SSS	− 15.8087	− 0.3162	LYS65, LYS31, TYR35, GLU48, VAL44, TYR140, TYR67, LEU143, ILE40, ILE77, TYR159, SER53, UNL1
RXP03_SSS_gluc_Ac	− 16.33468	− 0.2238	ALA55, VAL44, TYR159, LYS65, CYS64, LEU46, TYR35, SER53, LYS31, ALA157, ILE40, ILE41, PRO154

#### Role of glucose component

Glucose derivatives generally bind to claudins with lower docking energies, i.e., stronger binders with some exceptions, the *RRR*, *SRR,* and *SSS* compound docked to claudins 15 and 19. However, the difference is always small (Tables 1, 2, 3). In contrast, by the LE values, the unglycosylated compounds were unambiguously stronger binders than the corresponding glycosylated derivatives (Tables 1, 2, 3). The only case when the glycosylated derivative could be considered as a binder, showing the threshold LE value, is the compound with *RSR* configuration at claudin-15.

#### Role of the chirality of phosphorous

The chirality has no direct effect on the LE values. Thus, no conclusion can be drawn for binding preference. It should be noted, however, that these molecules all have enough flexibility to compensate for some unfeasible chirality, probably because none of them can be considered a strong binder (Tables 1, 2, 3).

#### Multivariate analysis of the residue-level interaction pattern

**Fig. 3:**
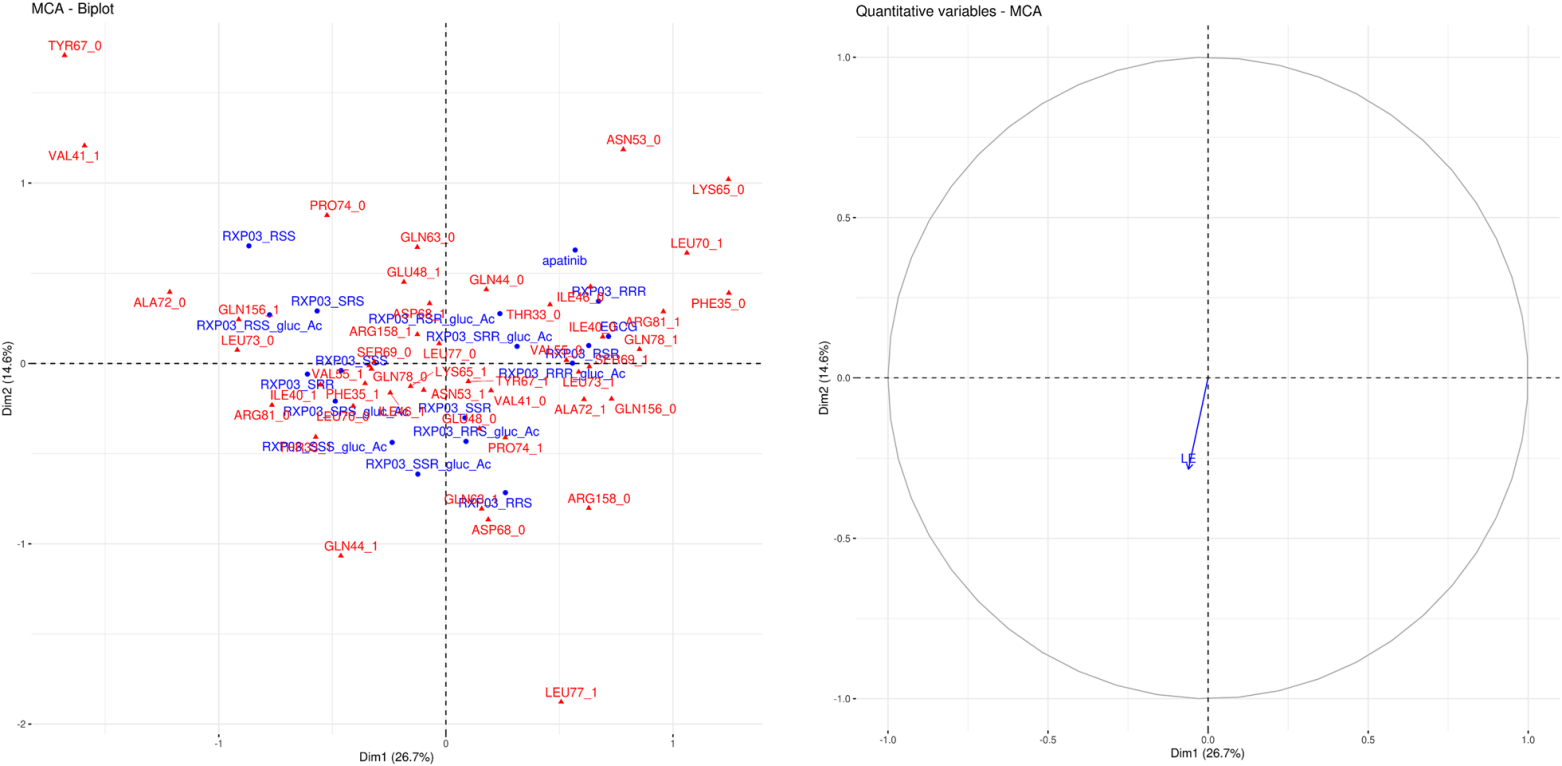
Biplot (left) of the interaction patterns and projection of the ligand efficiency on the factor map for claudin-4 (right). Interacting residues of the compounds are shown in red and blue, respectively

**Fig. 4:**
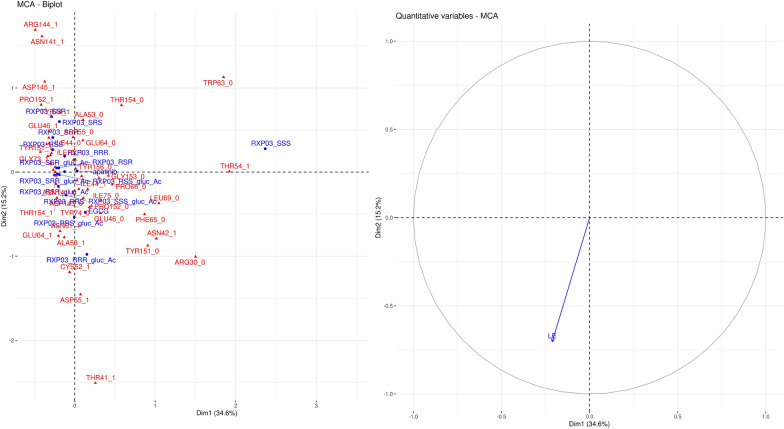
Biplot (left) of the interaction patterns and projection of the ligand efficiency on the factor map for claudin-15 (right). Interacting residues of the compounds are shown in red and blue, respectively

**Fig. 5:**
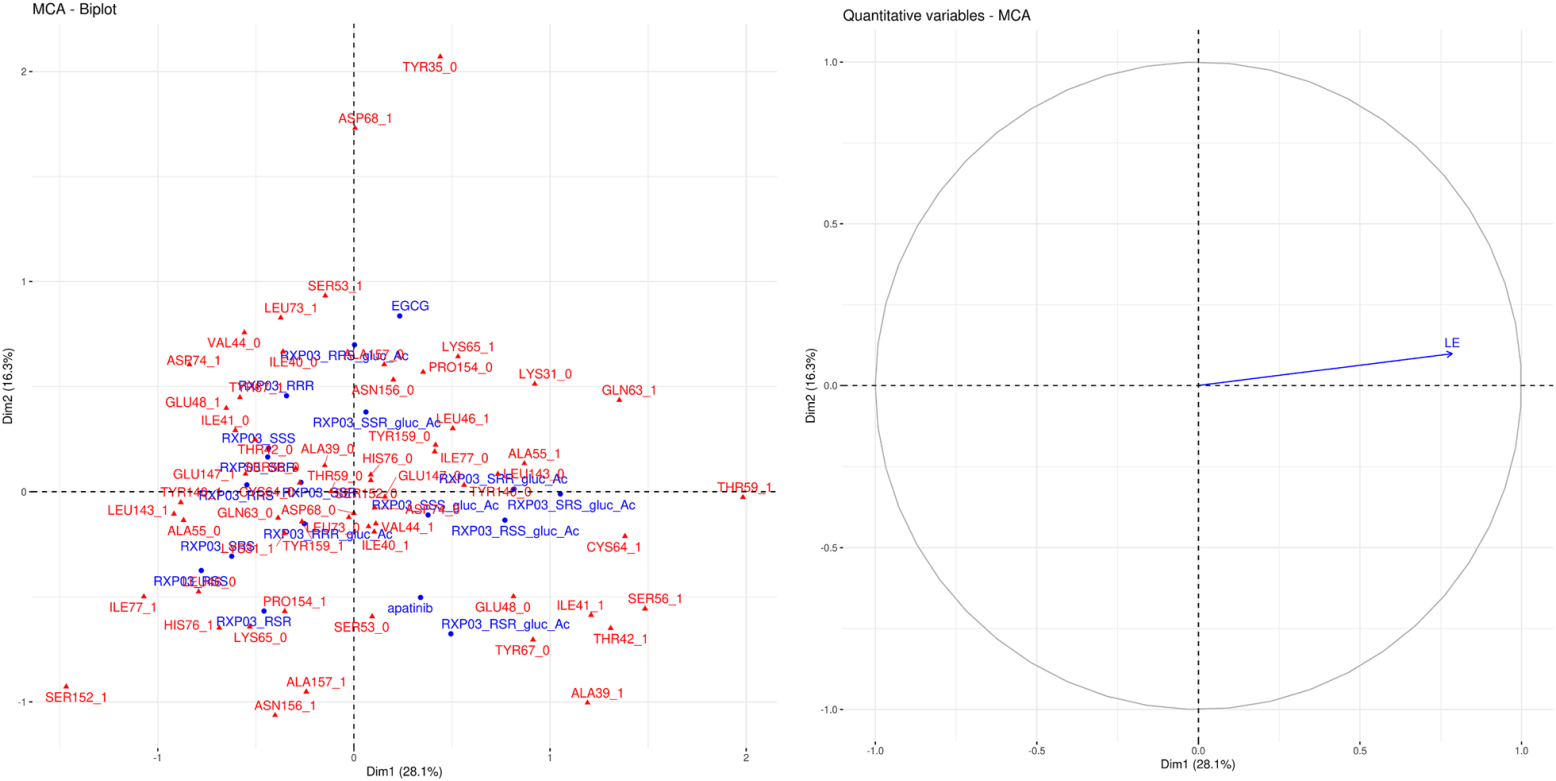
Biplot (left) of the interaction patterns and projection of the ligand efficiency on the factor map for claudin-19 (right). Interacting residues of the compounds are shown in red and blue, respectively

Instead of counting different interactions (polar, hydrophobic etc*.*) to compare, the full interaction patterns of the docked ligands were used for comparison. Although the current approach does not involve either chemical information about the type of the interactions like hydrogen bonding, aromatic-aromatic etc*.* or physical like attractive or repulsive, it holds information about the full list of the sites the interactions appeared at. This is presented as lists of the specific interacting residues of the protein in a tabular form inputted to MCA which can be seen in Tables 1, 2, 3. Multivariate statistics is a descriptive tool which can interpret this kind of data. The results are shown in biplots of the factor maps, which show relations between the interacting protein residues and the binding ligands (Figs. 3, 4, 5). Additionally, as a supplementary quantitative variable (not involved in the MCA analysis), the LE values were mapped onto the factor maps to add a direction for the energetically preferred residues and ligands within this series of ligands. The opposite direction of the LE values (deeper interaction energy) points to stronger binding.

**Fig. 6:**
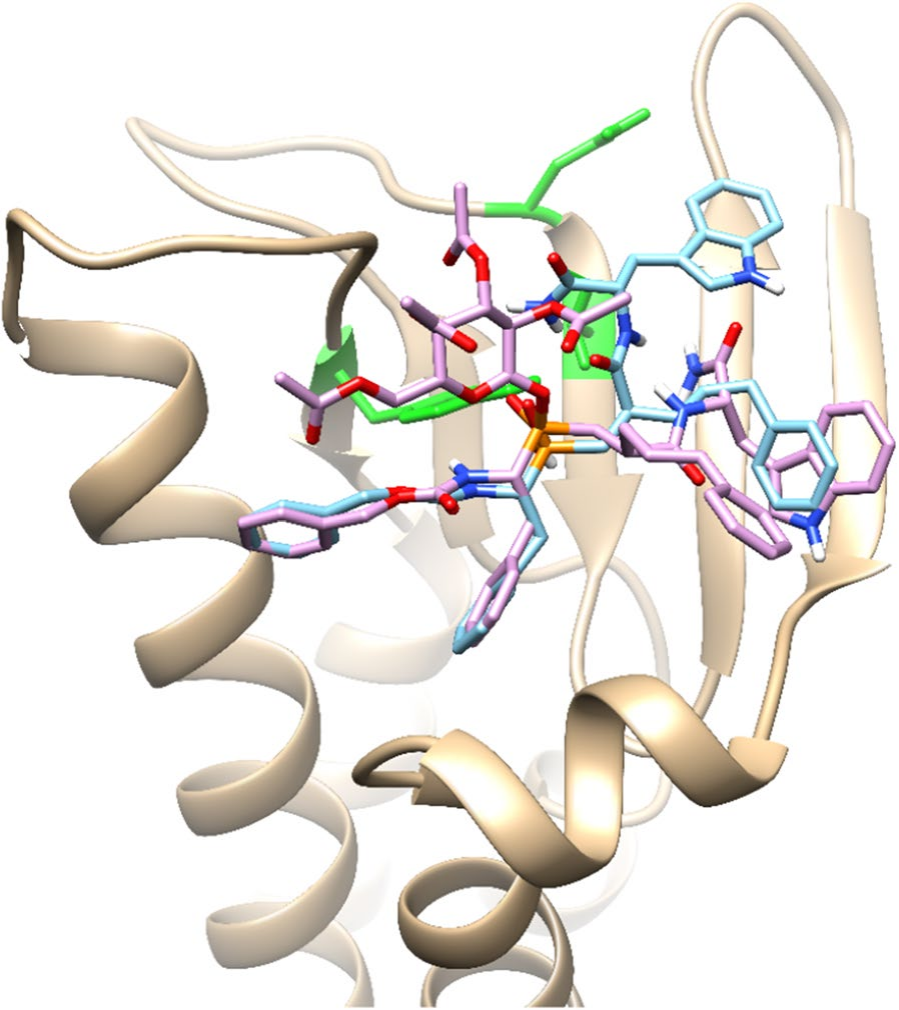
Docked poses of **RXP03**_RSR_gluc_(magenta) and Ac and **RXP03**_RSR (cyan) on human claudin-15. Interacting residues are shown in green

Although the factor maps do not show the separation of the ligands as binders or non-binders, it is notable that the opposite direction of the LE vector always points toward the stronger binders. However, accepting the binder/non-binder threshold value, the estimated values for the sugar-conjugated derivatives suggest that they are non-binders. Thus, an estimated interaction pattern could be meaningless. Based on these observations, the non-conjugated compounds could bind all three human claudins investigated, while the conjugated compounds would not. The only candidate can be the **RXP03**_RSR_gluc_Ac at claudin-15. The docked poses of the *RSR* compounds pair to claudin-15 (Fig. 6). Some of the interacting residues in the proximity of **RXP03**_RSR and **RXP03**_RSR_gluc_Ac on the biplot are also shown.

## Experimental section

### Detailed experimental procedures

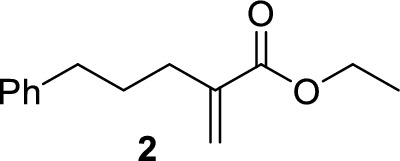



#### Ethyl-2-methylene-5-phenylpentanoate (2) [28]

A solution of *t-*BuOK (841 mg, 7.5 mmol) in dry DMF (25 ml) was added (C_2_H_5_O)_2_P(O)CH_2_CO_2_C_2_H_5_ **1** (1 ml, 5 mmol) slowly and the reaction mixture (RXM) was allowed to stir for 10 min at 10 0 °C under Ar. The C_6_H_5_(CH_2_)_3_Br (1.14 ml, 7.5 mmol) was added slowly into the flask, and the RXM was allowed to stir for 3 h at 85 °C under Ar. Then K_2_CO_3_ (2 g, 15 mmol) and paraformaldehyde (450 mg, 15 mmol) were added, and the resulting RXM was kept at reflux for 6 h. After completion, 0.5 M HCl was used to quench the reaction to pH ~ 5, and the RXM was extracted twice with Et_2_O. Drying the organic phase with MgSO_4_, then filtering and evaporating it under reduced pressure to afford pure **2** after purification of the residue by column chromatography (PE/AcOEt, v/v, 3/2) as a colorless oil (654 mg, 60%); ^1^H NMR (400 MHz, CDCl_3_) *δ* 1.29 (t, *J* = 7.12 Hz, 3H), 1.77–1.85 (m, 2H), 2.35 (m, 2H), 2.64 (m, 2H), 4.21 (q,* J* = 7.13 Hz, 2H), 5.52 (s, 1H), 6.15 (s, 1H), 7.16–7.19 (m, 3H), 7.24–7.29 (m, 2H); ^13^C NMR (100 MHz, CDCl_3_) *δ* 14.38, 30.10, 31.59, 35.43, 60.60, 124.48, 125.87, 128.43, 140.70, 142.15, 167.41.

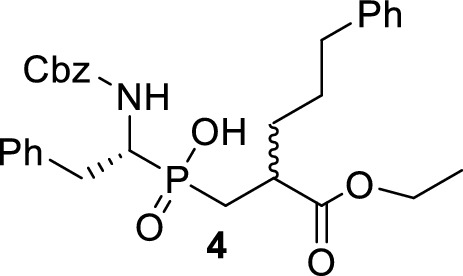



#### (*R, R/S*)-2-[(1-Benzyloxycarbonylamino-2-phenyl–ethyl)-hydroxy-phosphinoyl methyl]-5-phenyl pentanoic acid ethyl ester (4) [24]

A mixture of the phosphinic acid *(R)-3* (1.6 g, 5 mmol) and HMDS (5.3 ml, 25 mmol) was flushed with Ar and heated at 110 °C for 3 h. A dropwise addition of **2** (1.42 g, 6.5 mmol) was performed for 30 min, followed by stirring for 4 h. Once the RXM had cooled to 70 °C, absolute EtOH (6 ml) was added dropwise and stirred for 30 min at RT. Following concentration, the residue was dissolved in AcOEt, washed with 2 M HCl, brine, and dried over anhydrous Na_2_SO_4_, and the crude product was obtained after solvent evaporation. Flash chromatography using (DCM/MeOH/AcOH, v/v, 7/0.3/0.3) as eluent provided the title product **4** as a white solid (2.4 g, 90%). ^1^H NMR (400 MHz, CDCl_3_) *δ* 1.19–1.30 (m, 3H), 1.43–1.57 (m, 4H), 1.61–1.81 (m, 1H), 2.13–2.37 (m, 1H), 2.51–2.63 (m, 2H), 2.72–2.99 (m, 2H), 3.21–3.36 (m, 1H), 4.20–4.40 (m, 3H), 4.98 (s, 2H), 5.50–5.69 (m, 1H), 7.10–7.36 (m, 15H); ^13^C NMR (100 MHz, CDCl_3_) *δ* 14.21, 28.48, 28.66, 34.50, 35.10, 36.13, 39.49, 52.35, 67.84, 67.99, 126.21, 127.32, 127.88, 128.51, 128.88, 128.93, 129.10, 129.43, 129.67, 136.23, 136.54, 141.92, 156.25, 176.77; ^31^P NMR (162 MHz, CDCl_3_) *δ* 52.88, 53.76.

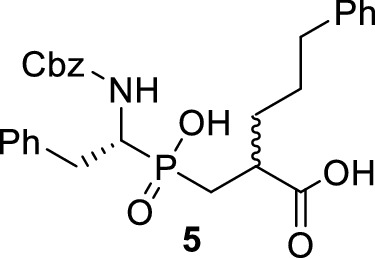



#### (*R,R/S*)-2-[(1-Benzyloxycarbonylamino-2-phenyl–ethyl)-hydroxy-phosphinoyl methyl]-5-phenyl-pentanoic acid (5) [24]

1 M NaOH_(aq)_ (100 ml) was added dropwise to a stirred solution of compound **4** (3.5 g, 6.5 mmol) in EtOH (100 ml). The RXM was allowed to stir for 24 h at RT. After removing the solvent, the residue was diluted with H_2_O, acidified with 3 M HCl in ice water to pH = 1, filtered, and washed with H_2_O. (3 × 10 ml), and Et_2_O (10 ml) and dried over P_2_O_5_ overnight afforded **5** as a white solid (3.1 g, 93%). ^1^H NMR (400 MHz, CDCl_3_) *δ* 1.44–1.56 (m, 4H), 1.65–1.88 (m, 1H), 2.13–2.30 (m, 1H), 2.52–2.66 (m, 2H), 2.87–2.88 (m, 2H), 3.11–3.37 (m, 1H), 4.25–4.39 (m, 1H), 4.98 (s, 2H), 5.38–5.63 (m, 1H), 7.12–7.29 (m, 15H); ^13^C NMR (100 MHz, CDCl_3_) *δ* 28.12, 28.90, 33.31, 34.21, 35.53, 38.59, 51.32, 68.21, 126.39, 127.52, 127.93, 128.49, 128.56, 128.79, 128.96, 129.27, 129.81, 136.40, 136.92, 141.92, 157.73, 181.55; ^31^P NMR (162 MHz, CDCl_3_) *δ* 51.65, 53.13.

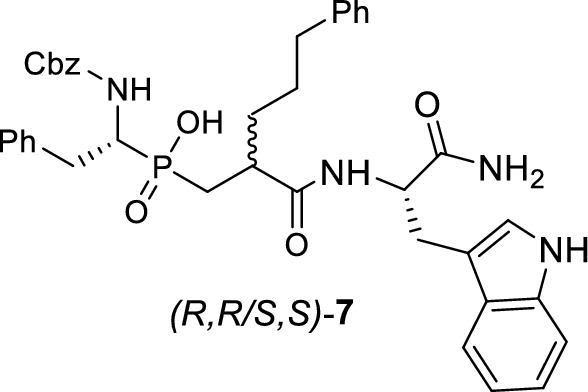



#### (*R*, *R/S*, *S*)(1-Benzyloxycarbonylamino-2-phenyl–ethyl)-{2-[1-carbamoyl-2-(1*H*-indol-3-yl)-ethylcarbamoyl]-5-phenyl-pentyl}phosphinic acid ((*R, R/S, S)-7)* [24]

To a chilled solution of **5** (2 g, 4 mmol) in DCM (90 ml) containing DIPEA (0.68 ml, 4 mmol), a solution of* S-*tryptophan amide **6** (0.8 g, 40 mmol), HOBt (0.52 g, 4 mmol), EDC.HCl (3.1 g, 16 mmol) and DIPEA (0.68 ml, 4 mmol) were added, and the RXM was allowed to stir for 30 min at 0 °C and then at RT overnight. After being finished, the RXM was diluted with DCM (200 ml), washed with a solution of 1 M HCl (2 × 10 ml), a saturated solution of NH_4_HCO_3_ (3 × 10 ml), 1 M HCl to pH = 1, and brine (30 ml). The organic layer was dried over anhydrous Na_2_SO_4_ and concentrated *in vacuo.* The residue was purified over silica gel chromatography using (DCM/MeOH/AcOH, v/v, 7/0.8/0.5) as eluent yielding (*R, R/S, S)-7 *as a white solid (2 g, 72%).

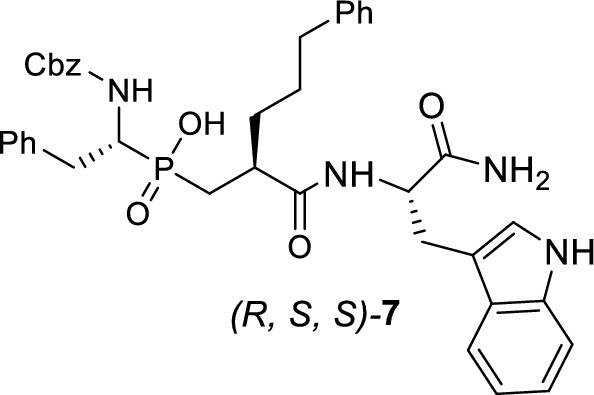



#### (*R*, *S*, *S*)(1-Benzyloxycarbonyl amino-2-phenyl–ethyl)-{2-[1-carbamoyl-2-(1*H*-indol-3-yl)-ethyl carbamoyl]-5-phenyl-pentyl}phosphinic acid ((*R, S, S)-7)* [24]

A stirred solution of (*R,R/S,S)-7* (2 g, 2.87 mmol) in EtOH (50 ml) was refluxed for 30 min and then left at 4 °C overnight. After 18 h at this temperature, the white solid precipitate was filtered and washed with cold absolute EtOH and dried over P_2_O_5_ to give (*R,S,S)-7* (1.65 g, 83%). ^1^H NMR (400 MHz, DMSO-*d*_*6*_) *δ* 1.21–1.57 (m, 4H), 1.62–1.76 (m, 1H), 1.92–2.07 (m, 1H), 2.29–2.47 (m, 2H), 2.58–2.78 (m, 2H), 2.97–3.15 (m, 2H), 3.16–3.27 (m, 1H), 3.81–3.97 (m, 1H), 4.35–4.47 (d, 1H), 4.79–5.00 (m, 2H), 6.88–7.37 (m, 18H), 7.57–7.74 (m, 3H), 7.99 (d, 1H), 10.81 (s, 1H); ^13^C-NMR (100 MHz, DMSO-*d*_*6*_) *δ* 27.57, 28.54, 28.82, 29.43, 33.33, 34.15, 35.52, 52.58, 53.61, 54.15, 65.71, 111.22, 111.78, 118.61, 125.99, 127.40, 127.83, 127.97, 128.61, 128.61, 128.71, 129.42, 136.55, 137.69, 142.52, 156.34, 174.01, 174.12; ^31^P-NMR (162 MHz, DMSO-*d*_*6*_) *δ* 44.88, 44.15. HRMS (ESI/QTOF) *m/z:* [M+H]^+^ Calcd for C_39_H_43_N_4_O_6_PH 695.2998; Found 695.2988.

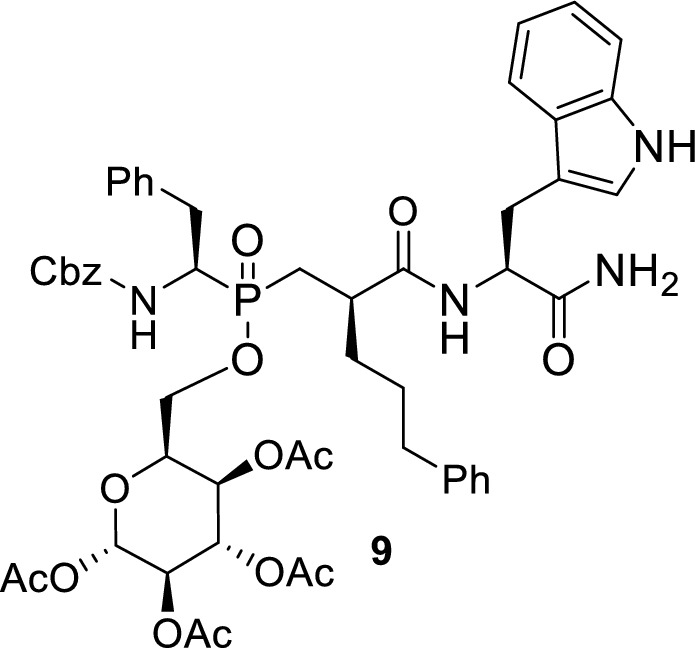



#### (*2S,3R,4S,5R,6S*)-6-(((((*R*)-2-(((*S*)-1-amino-3-(1*H*-indol-3-yl)-1-oxopropan-2-yl)carbamoyl)-5-phenylpentyl)((*R*)-1-(((benzyloxy)carbonyl)amino)-2-phenyl ethyl)phosphoryl)oxy)methyl)tetrahydro-2*H*-pyran-2,3,4,5-betrayal tetraacetate (9)

Thionyl chloride (0.36 g, 3 mmol) was added dropwise to the solution of (***R, S, S***)-***7*** (0.83 g, 1.2 mmol) in Et_2_O (5 ml) and 0.5 ml of DMF under nitrogen at 0 °C. The RXM was stirred at RT for 1.5 h and then concentrated. This resulting solid (0.80 g) dissolved in 7 ml of toluene, and the resulting solution was added dropwise to the mixture of **8** (0.42 g, 1.2 mmol) and TEA (0.2 ml, 1.43 mmol) in 5 ml of toluene at 0 °C. The RXM was refluxed for 9 h (monitored with ^31^P NMR), and then the triethylamine hydrochloride was removed by filtration. The filtrate was dried under vacuum, dissolved in Et_2_O and washed with a saturated solution of NaHCO_3_ and brine. The organic phase was dried over MgSO_4_ and filtered. The filtrate was concentrated and then treated with Et_2_O/hexane (1/1) at 0 °C, the white solid precipitate was filtered off and dried to give phosphinate **9** (0.94 g, 77%); ^1^H NMR (400 MHz, DMSO-*d*_*6*_) *δ* 1.19–1.42 (m, 4H), 1.60–1.72 (m, 1H), 1.86 (s, 3H),1.97–2.07 (m, 1H), 2.13 (s, 3H), 2.21 (s, 3H), 2.32 (s, 3H), 2.49–2.57 (m, 2H), 2.71–2.88 (m, 2H), 2.96–3.11 (m, 2H), 3.26–3.35 (m, 1H), 3.49–3.67 (m, 2H), 3.83–4.00 (m, 1H), 4.36–4.49 (d, 1H), 4.74–5.01 (m, 2H), 5.13 (m, 2H), 5.30–5.51 (t, 1H), 5.79–5.91 (d, 1H), 6.91–7.32 (m, 18H), 7.51–7.70 (m, 3H), 7.80–7.90 (d, 1H), 10.68 (s, 1H);^13^C-NMR (100 MHz, DMSO-*d*_*6*_) *δ* 21.11, 21.32, 21.44, 21.69, 27.23, 28.45, 28.34, 30.35, 33.01, 34.25, 35.61, 53.16, 54.34, 54.98, 60.25, 64.88, 68.12, 70.38, 72.67, 75.55, 91.51, 111.79, 111.89, 119.62, 125.03, 128.20, 128.33, 128.54, 128.61, 128.73, 128.89, 129.32, 136.11, 137.21, 143.50, 157.34, 168.13, 168.45, 168.65, 170.11, 174.36, 174.88; ^31^P-NMR (162 MHz, DMSO-*d*_*6*_) *δ* 49.01, 49.95. HRMS (MALDI) *m/z:* [M]^+^ Calcd for C_53_H_61_N_4_O_15_P 1024.3871; Found 1024.3858.

### Virtual screening

#### Molecular docking

Docking calculations were performed to the available crystal structure of human claudin-4 (PDB code 5B2G, chain C), and the homology models of human claudin-15 and claudin-19 based on the corresponding mouse crystal structures (PDB codes and protein chains used were 4P79, chain A and 3X29, chain A, respectively). The docking program PLANTS [29] was chosen due to its built-in chemical capabilities. PSOVina [30] was used to rescore the docking energies of the docked poses obtained by PLANTS. PSOVina calculated the docking energies in kcal/mol, also the ligand efficiency values [27] and listed the interacting receptor atoms and residues. The centre of the docking sphere (the search space) was set to the centre of the putative interacting surface of ECL1 for each claudin protein. The docking method was similar to [31], i.e. the efficiency of the stochastic search was improved by repeating the calculations five times [32], and the docking with the lowest energy was regarded as the final result. Furthermore, the size of the docking sphere was calculated for each ligand individually in the program SPORE [29] based on the idea in [33]. However, the size of the docking shere was increased by 5 angstroms due to the uncertainness of the optimal centre and to allow the ligands more freedom to find the best fit.

#### Preparation of the ligands

The 3D structures of the ligands were drawn by the molecular structure editor program Avogadro [34]. The structures of apatibin and EGCG were downloaded from PubChem for further use. The protonation state of the molecules was set to pH 7.4, and then the structures were energy minimized using the MMFF94s molecular force field and conjugate gradient method. The minimization was terminated at 10^–10^ kcal/mol energy gradient.

#### Preparation of the protein structures

Residues missing in the crystal structures of human claudins were reconstructed by the homology modelling program MODELLER v10.2 [35], leaving the crystal structure's atomic coordinates unchanged. The 3D structure of human claudins 15 and 19 was prepared by MODELLER using the corresponding mouse crystal structures (PDB codes and protein chains used were 4P79, chain A, and 3X29, chain A, respectively. The human claudin 15 and 19 amino acid sequences were downloaded from Uniprot with accession codes P56746 and Q8N6F1, respectively.

#### Comparison of the interaction patterns of the docking poses by multivariate statistics

Docking results were evaluated by calculating docking energies and the poses the contacting receptor atoms characterized. The latter, a list of amino acid residues for each ligand, was compared by multiple correspondence analysis (MCA) using the R programming environment [36, 37]. The lists of the residues contacting the ligands were summarized. Each docking pose was characterized by a binary vector showing which residue in the list was involved in the interaction. These binary vectors, as interaction patterns, were used by MCA to compare the compounds. The biplots visualized the results in the factor maps showing the compounds belonging to the specific interactions. A molecular graphic was prepared by the molecular visualizer program Chimera v. 1.15 [38].

## Conclusion

Quite often, esters can enhance the absorption and oral drug delivery of parent drugs. Considering these, various known drugs in clinical trials were discovered using esterification, such as sultamicillin, benorilate, and phosphinic inhibitor of ACE fosinoprilat. To find a novel MMP-11 prodrug, a novel phosphinate was developed by modifying the **RXP03** structure to synthesize a novel compound, **RXP03-sugar** ester. Based on the docking results, none of the structural features alone could be a main driving force of stronger binding to the claudins investigated. Overall, the glucose derivatives would bind to claudins less preferably than their parent compounds and, regarding the mainly hydrophobic nature of the ECL1 of claudins (Figs. 3, 4, 5), it happens instead through hydrophobic interactions. A minor decrease caused by the glucose moiety was observed in the case of Claudine 19. It may be concluded that claudin 19 can bind the glucose derivatives with almost the same strength as the parent compound, while claudins 4 and 15 bind it significantly weaker. Due to the importance of **RXP03-sugar** esters, the stability of the prodrug in simulated gastric, intestinal, and plasma fluid, its solubility in vitro, and PAMPA-BBB studies will also be investigated and reported separately.

## Data Availability

All data generated or analysed during this study are included in this published article.
